# Validation of DIABSCORE in screening for Type 2 Diabetes and prediabetes in Tunisian population

**DOI:** 10.1371/journal.pone.0200718

**Published:** 2018-08-15

**Authors:** Fadoua Gannar, María del Cristo Rodriguez-Pérez, Santiago Domínguez Coello, Khedija Haouet, Buenaventura Brito Díaz, Antonio Cabrera de León

**Affiliations:** 1 Research Unit ‘Integrated Physiology’, Laboratory of Biochemistry-Human Nutrition, Faculty of Sciences of Bizerte, UR11ES33 Carthage University, Tunis, Tunisia; 2 Primary Care Research Unit and University Hospital Nuestra Señora de Candelaria, Tenerife, Spain; 3 La Victoria Health Center, Tenerife, Spain; 4 Laboratory of Biochemical Analysis, University Hospital Mohamed Taher Maamouri, Nabeul, Tunisia; 5 Department of Preventive Medicine, La Laguna University, Tenerife, Spain; Florida International University Herbert Wertheim College of Medicine, UNITED STATES

## Abstract

**Aims:**

To perform a validation of DIABSCORE in a sample of Tunisian adults and find out the optimal cut-off point for screening of Type 2 diabetes (T2D) and prediabetes.

**Methods:**

225 adults 18–75 years and a subgroup of 138 adults (18–54 years), with undiagnosed T2D from the region of Cap-Bon, Tunisia were included in the present study. The DIABSCORE was calculated based on: age, waist/height ratio, family history of T2D and gestational diabetes. Receiver operating characteristics (ROC) curves and areas under curve (AUC) were obtained. The T2D and prediabetes prevalences odds ratios (OR) between patients exposed and not exposed to DIABSCORE≥90 and DIABSCORE≥80, respectively were calculated in both age ranges.

**Results:**

For screening of T2D the best value was DIABSCORE = 90 with a highest sensitivity (Se), negative predictive value (NPV) and lower negative likelihood ratio in participants aged 18–75 yr (Se = 97%; NPV = 97%) when compared to participants aged 18–54 yr (Se = 95%; NPV = 97%); for prediabetes, the best Se and NPV were for DIABSCORE = 80 in both age groups, but it showed a disbalanced sensitivity-specificity. The ROC curves for T2D showed a similar AUC in both age ranges (AUC = 0.62 and AUC = 0.61 respectively). The ROC curves for prediabetes showed a highest AUC in those aged 18–54 years than the older ones (AUC = 0.62 and AUC = 0.57, respectively). The prevalences OR of T2D for DIABSCORE≥90 was higher than for DIABSCORE≥80 in both age ranges. Nevertheless, the prevalences OR of prediabetes for DIABSCORE≥90 was half of the detected for DIABSCORE≥80 in both age ranges.

**Conclusion:**

The DIABSCORE is a simple clinical tool and accurate method in screening for T2D and prediabetes in the adult Tunisian population.

## Introduction

Diabetes mellitus (DM) is a major risk factor for cardiovascular disease [1]. Diabetes mellitus is one of the largest global health emergencies of the 21st century [2]. Type 2 Diabetes (T2D) is the most frequent type and accounts for about 90–95% of all diagnosed cases of DM [2]. The global prevalence of T2D is 8–9% [3] and has been rising more rapidly in middle- and low-income countries.

Patients with T2D have a higher risk of death from cardiovascular causes compared with their nondiabetic counterparts. The WHO projected that diabetes will be the seventh leading cause of death in 2030 [4] and the mortality rate of DM associated cardiovascular disease is different among ethnic groups and sexes groups [5]. Rates of T2D are speedily growing worldwide, and a number of risk factors contribute to incident diabetes, most prominently age, family history, and obesity [6, 7]. Evidence exits that early detection of established diabetes improves outcome and reduces the incidence of the disease in people with glucose abnormalities, even since prediabetes status has been established.

In spite of the high prevalence of diagnosed T2D, almost half of all people with the disease are unaware of their disease [5]. The awareness of T2D increases with age, economic level and it was higher amongst those with family history of T2D for both genders [8]. In view of the economic burden of diabetes and its associated comorbidities, a public health policy aiming to target the major risk factors in each population might be more effective in preventing diabetes. Diabetes frequently remains undiagnosed in developing countries [9]. Nevertheless, Tunisia a developing nation is at the cusp of a health-care transition. Health-care facilities are improving across the country. However, the prevalence of T2D in Tunisia was 15.1% in 2014 [8].

Given the importance of the problem, more than a hundred of clinical-epidemiological risk assessment tools have been developed to identify subjects at high risk of T2D, but limited evidence exits for the use of these tools as part of a health policy or clinical practice guideline. Diabetes risk assessment tools generally comprise a set of questions about diabetes risk factors combined with several straightforward measurements of anthropometric indexes to calculate a risk score and many include invasive approaches. All of which makes them expensive and impracticable in real conditions of clinical practice [10]. Most of these tools have shown difficulties when they are validated in different populations or ethnicities. A risk score for screening of T2D (DIABSCORE) was developed and validated in a general population cohort from Canary Islands [11]. Also, the DIABSCORE was validated in patients undergoing primary care in 2 regions of Spain, and the results have shown that it is cost-effective and applicable method in screening for T2D [12]. DIABSCORE reached out, in both genders, a high sensitivity to detect cases of T2D and high capacity to discriminate (NPV), providing a fast and reliable method to reject the presence of diabetes with a high satisfaction of professionals and patients. In addition, DIABSCORE includes noninvasive tests, and requires only information about age, height, waist circumference, and family history of diabetes or gestational diabetes.

The aim of our study, was to perfom an external validation of DIABSCORE in a sample of Tunisian adults and to investigate the optimal cut-off point in this population for screening of T2D and prediabetes.

## Materials and methods

### Design and subjects

A cross-sectional study was conducted during the year 2016 in adults from general population of the region of Nabeul, northeastern Tunisia. In total 300 subjects were randomly recruited from the general population census, and they were aged between 18 and 75 years. All the objectives and procedures were explained in writting to the participants and all of them signed the informed consent. The Clinical Research Ethics Committee of the University Hospital Mohamed Taher Maamouri approved the study protocol (03/2014). Exclusion criteria were: previous diagnosis of T2D mellitus, participants with antidiabetic treatment, pregnant women, patients with iron metabolism disorders or hemoglobinopathies. The final number of participants in this study was 225.

The necessary data to calculate DIABSCORE was: age in years, waist circumference in cm (measured using a non stretchable tape with the patient in light clothing and normal breathing, at the midpoint between the iliac crest and the last rib), height in cm (with patient standing and barefoot), family history of T2D in first-degree relatives younger than 65 years, and for women, personal history of gestational diabetes. With the collected data, DIABSCORE was computed automatically as detailed in its previous validation report [11], each variable was given the following score: age (the number of years), waist-to-height-ratio (the value of the waist-to-height-ratio multiplied by 100), family history of T2D (Yes = 10 points, No = 0 points), and history of gestational diabetes (Yes = 25 points, No = points).

After at least 10 hours of fasting, venous blood samples were drawn for measurement of baseline glucose (mg/dL) and HbA1c (%). These tests were performed in reference laboratory of the University Hospital Mohamed Taher Maamouri Hospital-Nabeul, meeting national and international quality standards. When glucose was ≥ 126 mg/dl (7 mmol/L), participants were considered as having undiagnosed T2D. Prediabetes was defined as a glucose level ≥ 100 mg/dl (5.6 mmol/L) and ≤ 125mg/dL (6.9 mmol/L).

### Statistical analysis

The age range for which DIABSCORE initially showed the highest safety levels was 18–54 years in the Spanish population. In our study (n = 225), the participants aged 18–54 years were only 138. Despite the small size, we validated DIABSCORE in both age ranges (S1 File).

The continuous variables were summarized by means ± standard deviations and the categorical variables as proportions (%) with confidence interval of 95%. For independent samples when two means were compared, *Student's test* was used. If the variables were not normal, the *Mann-Whitney U test* was used. *Pearson's Chi Square* was used to compare proportions.

ROC curves were plotted using DIABSCORE as a contrast variable, with undiagnosed T2D or prediabetes as state variables. The areas under the curve (AUC) were evaluated and the cut-off points were obtained. The optimal cut-off value of DIABSCORE for screening of T2D and prediabetes was extrapolated and their predictive values were calculated.

The Open-Epi application (http://www.openepi.com) was used to compute the prevalences odds ratios, and to compare the excess risk of T2D or prediabetes among participants exposed and not exposed to values equal or greater than the cut-off point of the screening variable. The analyses were carried out with SPSS software, version 21.0.

## Results

The study comprised 225 without previously diagnosed T2D adults (18–75 years), with 138 adults aged between 18–54 years. The distribution of the main variables in both ranges of age was shown in **Table 1**. The mean age of participants was 49.9±16.5 and 39.1±10.2 respectively; With higher percentage of females, almost a half of participants presented overweight or obesity, and the prevalence of blood glucose ≥126mg/dL was 16% in the study population but when looking specifically at the age group 18–54 yr only, 13.8% of the individuals were above the cutt off for T2D. When we considered prediabetes, the prevalence was higher in both age groups (24.4% and 21% respectively). Nevertheless, all of them presented HbA1c values less than 6.5%. The frequency of a family history of T2D was high (26.7% in the 18–75 years participants and 33.3 in 18–54 ones). When the considered value of the DIABSCORE was ≥100, the prevalence of participants at risk of developing T2D was of 76.9% and 62.3% in the youngest group.

**Table 1:** General characteristics of the sample.

		Aged 18–75 yr (n = 225)	Aged 18–54 yr (n = 138)
		Mean ± SD	Mean ± SD
**Age (years)**		49.9 ± 16.5	39.1 ± 10.2
**Weight (kg)**		70.2 ± 14.3	70.9 ± 13.7
**Height (m)**		162.7 ± 9.2	162.7 ± 9.3
**BMI (kg/m**^**2**^**)**		26.5 ± 5.2	26.7 ± 4.9
**Waist circumference (cm)**		96.4 ± 13.0	95.3 ± 12.2
**Waist-to-height ratio**		59.4 ± 8.7	58.7 ± 8.3
**Blood glucose (mg/dL)**		107.9 ± 49.2	105.7 ± 49.0
**HbA1C (%)**		5.8 ± 1.3	5.7 ± 1.5
		**% (95% CI)**	**% (95% CI)**
**Gender**	**Men**	45.8 (39.3–52.3)	33.3 (25.4–41.2)
	**Women**	54.2 (47.7–60.7)	66.7 (58.8–74.6)
**BMI**	**BMI < 25**	44.1 (37.6–50.6)	41.9 (33.7–50.1)
	**BMI (25–29.9)**	26.6 (20.8–32.4)	27.2 (19.8–34.6)
	**BMI ≥ 30**	29.3 (23.4–35.2)	30.9 (19.8–34.6)
**Family history Type 2 diabetes**		26.7 (20.9–32.5)	33.3 (25.4–41.2)
**History gestational Diabetes**		7.1 (3.7–10.5)	11.6 (6.3–16.9)
**DIABSCORE Value** ≥**100**		76.9 (71.4–82.4)	62.3 (54.2–70.4)
**HbA1c (%)** ≥ **6.5%**		0.0 (0.0–0.0)	0.0 (0.0–0.0)
**Prediabetes**		24.4 (18.8–30.0)	21.0 (14.2–27.8)
**Type 2 Diabetes**		16.0 (11.2–20.8)	13.8 (8.0–19.5)

Prediabetes: Fasting blood glucose ≥100mg/dL and ≤125 mg/dL. Type 2 diabetes: Fasting blood glucose ≥126 mg/dL.

**Fig 1:**
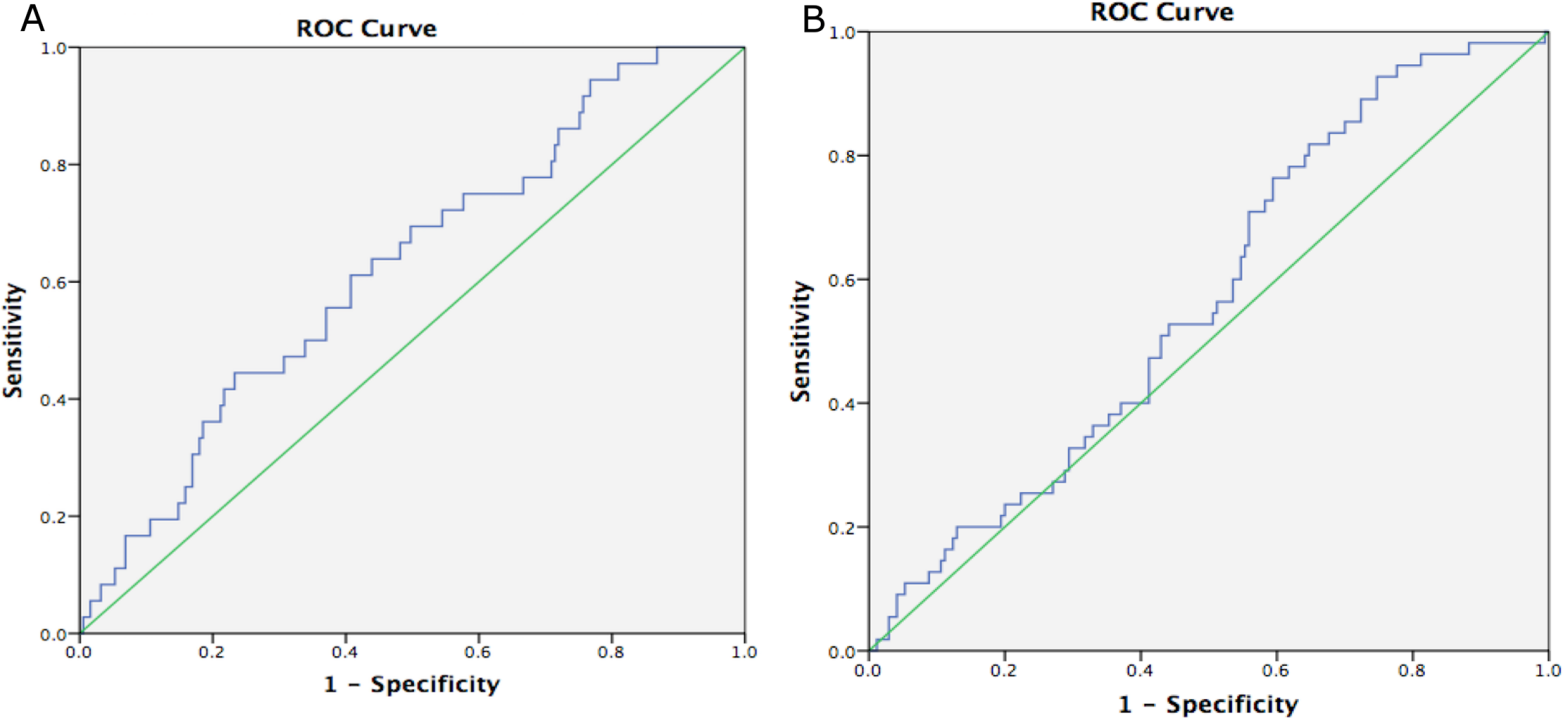
(A). ROC Curves and Area Under Curve (AUC) for DIABSCORE for detecting prevalence of undiagnosed diabetes according to fasting blood glucose ≥ 126 mg/dL, in participants aged 18–75 yr. AUC = 0.62 (0.53–0.72). (B). ROC Curves and Area Under Curve (AUC) for DIABSCORE for detecting prevalence of undiagnosed prediabetes according to ADA criteria (Blood glucose ≥100 and ≤125mg/dL), in participants aged 18–75 yr. AUC = 0.57 (0.49–0.65).

**Fig 2:**
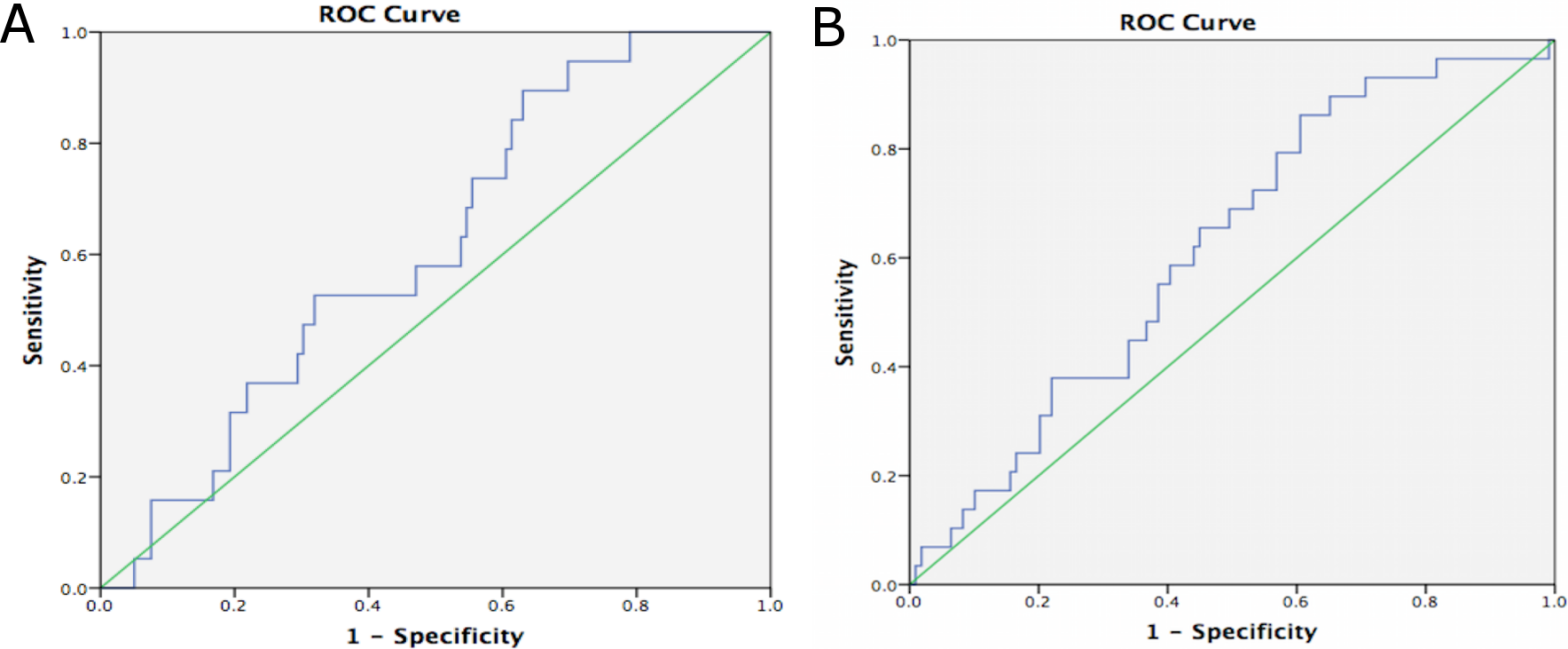
(A). ROC Curves and Area Under Curve (AUC) for DIABSCORE for detecting prevalence of undiagnosed diabetes according to fasting blood glucose ≥ 126 mg/dL, in participants aged 18–54 yr. AUC = 0.61 (0.49–0.73). (B). ROC Curves and Area Under Curve (AUC) for DIABSCORE for detecting prevalence of undiagnosed prediabetes according to ADA criteria (Blood glucose ≥100 and ≤125mg/dL), in participants aged 18–54 yr. AUC = 0.62 (0.51–0.72).

The ROC analysis for undiagnosed T2D (glucose≥126mg/dL) and for prediabetes (glucose ≥100mg/dL and ≤ 125mg/dL) and in both age ranges are shown in **Figs 1 and 2**respectively. In participants aged between 18–75 years, the AUC for DIABSCORE for T2D was higher than that obtained for prediabetes (0.62, CI_95%_ = 0.53–0.72 vs 0.57, CI_95%_ = 0.49–0.65). When we did the same analysis but in the 18–54 years range, the AUC were very similar for T2D and prediabetes.

The optimal cut-off points of DIABSCORE for screening of type 2 diabetes and prediabetes in the studied population were determined and their predictive values and likelihood ratios calculated. **Table 2**shows these results for age range of 18–75 years and in **Table 3**appears the same analysis in participants aged between 18–54 years. We note that the DIABSCORE cut-off point ≥90 for prediabetes and T2D, presented a very high sensitivity (96% and 97%, respectively) and, in a similar way, a very high negative predictive value (99% and 97%, respectively) and a negative likelihood ratios less than 0.5. In the case of participants aged between 18–54 years the cut-off for DIABSCORE = 90 obtained similar values than the older group ones, and it showed the most balanced sensitivity-specificity, a negative predictive value between 97–99% and a negative likelihood ratios less than 0.5 for prediabetes and T2D.

**Table 2:** Sensitivity, specificity, predictive values and likelihood ratios of DIABSCORE cut-off points in participants aged 18–75 yr.

**DIABSCORE Cut-off points for Prediabetes (Blood glucose ≥ 100 mg/dL and ≤125 mg/dL)**						
DIABSCORE Cut-off point	Sensitivity (%)	Specificity (%)	Positive predictive value (%)	Negative predictive value (%)	Positive Likelihood Ratio	Negative Likelihood Ratio
80	98	10	6	99	1.10	0.20
85	96	14	7	98	1.12	0.29
**90**	**96**	**18**	7	**99**	1.17	**0.22**
95	93	23	7	98	1.21	0.30
100	89	27	7	97	1.24	0.41
105	80	35	7	96	1.23	0.57
**DIABSCORE Cut-off points for Type 2 diabetes (Blood glucose ≥ 126 mg/dL)**						
DIABSCORE Cut-off point	Sensitivity (%)	Specificity (%)	Positive predictive value (%)	Negative predictive value (%)	Positive Likelihood Ratio	Negative Likelihood Ratio
80	100	9	16	100	1.10	0.00
85	97	13	16	96	1.12	0.23
**90**	**97**	**17**	17	**97**	1.17	**0.18**
95	94	22	18	95	1.21	0.27
100	86	25	17	91	1.15	0.56
105	78	33	17	89	1.16	0.67

**Table 3:** Sensitivity, specificity, predictive values and likelihood ratios of DIABSCORE cut-off points in participants aged 18–54 yr.

**DIABSCORE Cut-off points for Prediabetes (Blood glucose ≥ 100 mg/dL and ≤125 mg/dL)**						
DIABSCORE Cut-off point	Sensitivity (%)	Specificity (%)	Positive predictive value (%)	Negative predictive value (%)	Positive Likelihood Ratio	Negative Likelihood Ratio
80	97	16	7	99	1.15	0.19
85	93	22	7	98	1.19	0.32
**90**	**93**	**28**	8	**98**	1.29	**0.25**
95	86	37	8	98	1.37	0.38
100	79	42	8	97	1.36	0.50
105	62	55	8	96	1.38	0.70
**DIABSCORE Cut-off points for Type 2 diabetes (Blood glucose ≥ 126 mg/dL)**						
DIABSCORE Cut-off point	Sensitivity (%)	Specificity (%)	Positive predictive value (%)	Negative predictive value (%)	Positive Likelihood Ratio	Negative Likelihood Ratio
80	100	15	17	100	1.18	0.00
85	95	21	18	96	1.20	0.24
**90**	**95**	**28**	19	**97**	1.32	**0.18**
95	90	35	20	95	1.38	0.29
100	74	60	25	93	1.85	0.43
105	58	53	18	88	1.21	0.79

**Tables 4 and 5** show the prevalences odds ratios (OR) for prediabetes and T2D among participants exposed to DIABSCORE value ≥80 and DIABSCORE value ≥90. The participants aged 18–75 yr **(Table 4)** and exposed to DIABSCORE value ≥80 had higher OR, sensitivity and negative predicitive value for prediabetes than those exposed to value ≥90, while for T2D the OR was higher in those exposed to DIABSCORE value ≥90. The same situation was found between the participants aged 18–54 years **(Table 5)**.

**Table 4:** Prevalences odds ratios (OR) and 95% CI among participants aged 18–75 years exposed or not exposed to: DIABSCORE value ≥ 80 or DIABSCORE value ≥ 90 or prediabetes (blood glucose ≥100 and ≤125mg/dL) or type 2 diabetes (blood glucose ≥126 mg/dL).

	Glucose < 100 mg/dL	Prediabetes	Glucose < 126mg/dL	T2D
DIABSCORE <80	17	1	18	0
DIABSCORE ≥80	117	90	171	36
	p = 0.002 OR = 13.1 (CI_95%_ = 1.7–100.1) Se = 98.9 (CI_95%_ = 94–99.8) Sp = 12.7 (CI_95%_ = 8.1–19.4) NPV = 94.4 (CI_95%_ = 74.2–99) PPV = 43.5 (CI_95%_ = 36.9–50.3)		p = 0.054 OR = 1.2 (CI_95%_ = 1.1–1.3) Se = 100 (CI_95%_ = 90.4–100) Sp = 9.5 (CI_95%_ = 6.1–14.5) NPV = 100 (CI_95%_ = 82.4–100) PPV = 17.4 (CI_95%_ = 12.8–23.1)	
DIABSCORE <90	31	3	33	1
DIABSCORE ≥ 90	103	88	156	35
	p<0.001 OR = 5.2 (CI_95%_ = 1.7–15.5) Se = 96.7 (CI_95%_ = 90.7–98.9) Sp = 23.1 (CI_95%_ = 16.8–30.9) NPV = 91.2 (CI_95%_ = 77.0–96.9) PPV = 46.1 (CI_95%_ = 39.1–53.1)		p = 0.024 OR = 6.2 (CI_95%_ = 0.8–43.9) Se = 97.2 (CI_95%_ = 85.8–99.5) Sp = 17.5 (CI_95%_ = 12.7–23.5) NPV = 97.1 (CI_95%_ = 85.1–99.5) PPV = 18.3 (CI_95%_ = 13.5–24.4)	

OR: prevalence odds ratio, Se: sensitivity (%), Sp: Specificity (%). NPV: negative predictive value (%), PPV: positive predictive value (%). T2D: Type 2 diabetes.

**Table 5:** Prevalences odds ratios (OR) and 95% CI among participants aged 18–54 years exposed or not exposed to: DIABSCORE value ≥ 80 or DIABSCORE value ≥90 or prediabetes (blood glucose ≥100 and ≤125mg/dL) or type 2 diabetes (blood glucose ≥126 mg/dL).

	Glucose <100mg/dL	Prediabetes	Glucose < 126mg/dL	T2D
DIABSCORE <80	17	1	47	5
DIABSCORE ≥80	73	47	72	14
	p = 0.005 OR = 10.9 (CI_95%_ = 1.4–85.0) Se = 97.9 (CI_95%_ = 89.199.6) Sp = 18.9 (CI_95%_ = 12.1–28.2) NPV = 94(CI_95%_ = 74.2–99) PPV = 39.2 (CI_95%_ = 30.9–48.1)		p = 0.069 OR = 1.2 (CI_95%_ = 1.1–1.3) Se = 100 (CI_95%_ = 83.2–100) Sp = 15.1 (CI_95%_ = 9.8–22.6) NPV = 100 (CI_95%_ = 82.4–100) PPV = 15.83 (CI_95%_ = 10.4–23.4)	
DIABSCORE <90	31	3	33	1
DIABSCORE ≥90	59	45	86	18
	p = 0.000 OR = 5.2 (CI_95%_ = 1.7–15.5) Se = 96.7 (CI_95%_ = 90.7–98.9) Sp = 23.1 (CI_95%_ = 16.8–30.9) NPV = 91.2 (CI_95%_ = 77.09–96.9) PPV = 46.1 (CI_95%_ = 39.1–53.1)		p = 0.035 OR = 6.2 (CI_95%_ = 0.8–43.9) Se = 97.2 (CI_95%_ = 85.8–99.5) Sp = 17.5 (CI_95%_ = 12.7–23.5) NPV = 97.1 (CI_95%_ = 85.1–99.5) PPV = 18.3 (CI_95%_ = 13.5–24.4)	

OR: prevalence ratio, Se: sensitivity (%), Sp: Specificity (%). NPV: negative predictive value (%), PPV: positive predictive value (%). T2D: Type 2 diabetes

## Discussion

Our results show that DIABSCORE is a useful screening tool to discriminate risk of T2D and prediabetes in a Tunisian population. This score has been analyzed with 2 cut-off points (DIABSCORE ≥90 and DIABSCORE ≥80) and validated for different age ranges; in both situations it was effective and safe method with a high sensitivity and negative predictive value.

Over the last few decades, many screening scores for T2D have been developed, but they are not feasible in clinical practice. Some limitations in their uses are, the invasive tests that they include, the number of difficulties to extrapolate their results to other populations that they present, and the variables needed for their calculation, promote barriers in their general use [10]. The DIABSCORE is based only on age, waist-to-height ratio, family history of T2D, and history of gestational T2D, but without requiring any analysis of blood glucose or HbA1c, which made its cost-effectiveness very good. Moreover, the DIABSCORE could be managed by the nursing staff or even self-administrated, avoiding the time-consumption by the physicians. When DIABSCORE was tested in a primary care setting it showed a high degree of satisfaction and acceptance by both patients and professionals, finding in two different Spanish populations a high sensitivity and NPV for DIABSCORE ≥100 [12].

Glycemia and T2D are rising globally [6]. T2D is a main cause of morbidity, mortality, and health-system costs in the world [13, 14]. This growth is associated with increasing urbanization, economic development, ageing population, reduced physical activity and reduced healthy diets. In a previous study carried out between a developed country (Spain) and a developing one (Tunisia), showed a very rapid spread of the western pattern of chronic diseases towards developing countries [15]. In fact, in Tunisia ageing population together with social, economic and lifestyle changes have led to a dramatic increase in T2D [16]. The prevalence of T2D in Tunisia was 15.1% in 2014 (8), and a dramatic rise in prevalence by 2027 is estimated to 26.6% (28.6% in men and 24.7% in women) [17]. Thus, urgent measures are needed to prevent diabetes and its related complications.

Validating a risk model or score means testing its calibration and discrimination in a different setting. We focalize to validate the DIABSCORE in an adult sample from the general Tunisian population, in a similar way that the population in which DIABSCORE was developed and evaluated. We analyzed the cut-off points of the DIABSCORE as a clinical score for screening of T2D and prediabetes. Our findings demonstrate that DIABSCORE behaved as a good predictor for detecting diabetes risk as well as for detecting prediabetes, in both ranges of age. In contrast with the finding of the Spanish populations [12], in our case the optimal value of DIABSCORE for screening of T2D was ≥90, which presented better accurate levels than DIABSCORE ≥100. DIABSCORE showed some differences in relation to the age of the individuals. Despite the AUC for both, diabetes and prediabetes, showed a just moderate performance of DIABSCORE in all the participants, it was in the older group (18-75yr) where we found the lowest AUC for prediabetes screening. If we focus on the 95%CI AUC obtained, it could mean that DIABSCORE has shown a null discriminating power for T2D (in the age group of 18–54 years) and prediabetes (in 18–75 years group); nevertheless, when the confidence level was broadened to 90% (Figs 1 and 2), we found a better performance of DIABSCORE for T2D screening and also for prediabetes, with a 90%CI lower limit higher than 0.50 in all the cases.

In the original study in the cohort from Spain, the authors found out that the DIABSCORE obtained the highest safety levels in participants not older than 54 yr. When we analyzed the safety levels of DIABSCORE, we found that the cutoff point of 90 showed the most balanced sensitiviy-specificity values and a high negative predictive value. It also obtained a negative likelihood ratios (NLR) of less than 0.5, and very close to 0. The positive likelihood ratios (PLR) confirmed that DIABSCORE is not a good diagnostic test, with values less than 2. Given that DIABSCORE was designed and validated in Spanish studies as a screening tool to rule out T2D, we have found that the behavior of the test was similar in our sample that in the Spanish one. For screening of prediabetes in the group aged 18–54 years, the value ≥ 80 should be taken into account, but in this case, it presented a very disbalanced sensitivity-specificity. The prevalences odds ratios showed a high risk of T2D or risk of prediabetes when DIABSCORE was ≥90, more than for DIABSCORE ≥80. The highest OR and sensitivity and NPV or NLR value obtained showed that DIABSCORE ≥90 is the most appropriate method to rule out the risk of T2D in all participants.

We want to point out that our participants were older than the two Spanish-regions ones but they also presented a high prevalence of dysglucemia and abdominal obesity. Moreover, the reported prevalence of T2D in Tunisia is higher than the prevalence in Spain (8). Despite these important differences in the prevalences, we consider that we have validated and identified the optimal value of DIABSCORE in our population. Similarly, other screening diabetes tools have been validated in different populations, resulting in so different cut-off point. FINDRISK is the principal screening tool for diabetes testing in different populations but this screening method has often several limitations in relation to their applicability due to the variables involved [18–21]. DIABSCORE need neither complex questions nor invasive testing in comparison to other ones. In populations living in developing countries, special importance has the fact that no prior electronic health records or lifestyle information are required. Apart of this, the negative predictive value obtained allows individuals without risk to be excluded, not needing to perform a blood test with security levels of 97% or 99%.

To our knowledge, no previous studies have measured and validated the applicability of a risk assessment tool in identifying T2D or prediabetes in Tunisia. We recognise that our study has some limitations; the main weakness was the small sample size that we were able to recruit. If the studied sample had been larger, we had obtained better results about the discriminatory capacity of DIABSCORE as T2D screening tool. In this line, we could verify that with a lower confidence level (0.01), DIABSCORE identified the risk for undiagnosed cases of T2D and prediabetes. Another limitation was not being able to follow-up the studied population and to obtain incident cases of T2D that allow us to validate the DIABSCORE in them. Among the strengths, we should mention the validation of the DIABSCORE in the general population, aged between 18–75 years. Also, this screening tool is easy to apply and it is a cost-effectiveness method, as it was demonstrated previously [12].

## Supporting information

S1 File (SAV)
